# Ni(II) Interactions in Boreal *Paenibacillus* sp., *Methylobacterium* sp., *Paraburkholderia* sp., and *Pseudomonas* sp. Strains Isolated From an Acidic, Ombrotrophic Bog

**DOI:** 10.3389/fmicb.2019.02677

**Published:** 2019-11-26

**Authors:** Jenna Knuutinen, Malin Bomberg, Marianna Kemell, Merja Lusa

**Affiliations:** ^1^Department of Chemistry, University of Helsinki, Helsinki, Finland; ^2^VTT Technical Research Centre of Finland, Espoo, Finland

**Keywords:** nickel uptake, adsorption, sphagnum peat, heavy metal contamination, biopurification

## Abstract

The uptake of nickel [Ni(II)] by *Paenibacillus* sp., *Methylobacterium* sp., *Paraburkholderia* sp., and *Pseudomonas* sp. strains isolated from a boreal bog was studied using batch experiments. All strains removed Ni(II) from the solution and the uptake efficiency was affected by the nutrient source, incubation temperature, time, and pH. As highest Ni uptake (with a maximum K_d_ of 1890 L/kg DW) was recorded for the *Pseudomonas* sp. strains, these bacteria were used in the following protein expression (SDS-PAGE and MALDI-TOFF), transmission electron microscopy (TEM) and EDS experiments. In addition, Freundlich and Langmuir sorption isotherms were determined. In the Ni(II) treated cells, dense crystalline intra-cellular accumulations were observed in TEM examinations, which were identified as Ni accumulations using EDS. SDS-PAGE and MALDI-TOFF spectra of Ni(II) treated cells showed several changes in the protein profiles, which can indicate active accumulation of Ni in these bacteria. Concurrently, we observed Ni(II) uptake to follow Freundlich and Langmuir isotherms, suggesting straight cellular biosorption in addition to the intra-cellular accumulation. The role of cellular (cell membrane and cell wall) functional groups involved in Ni(II) binding were therefore studied using Fourier transformation infrared spectroscopy. These analyses supported the potential role of the alcoholic hydroxyl, carboxyl and amine groups in Ni(II) binding in these bacteria, therefore suggesting two different Ni(II) uptake mechanisms; (i) intra-cellular accumulation [possibly connected to detoxification of Ni(II)], and (ii) straight biosorption on cell membrane/wall functional groups.

## Introduction

Nickel (Ni) occurs extensively in Earth’s crust and is found in various mineral forms (e.g., pentlantide) (Cempel and Nikel, 2006). Significant amounts of Ni are released into the environment as a result of human activities, including mining, energy production (combustion of coal, diesel, etc.) and industrial use of Ni (e.g., production of ferronickel) (Nriagu, 1990). Industrial processes generally increase Ni mobility and may result in potentially large releases to the environment through surface and groundwater flows. In addition, the potentially high mobility of the long-lived Ni radioactive isotopes, ^59^Ni and ^63^Ni, found in spent nuclear fuel, are of concern for the long-term safety of spent nuclear fuel bedrock disposal (Hjerpe et al., 2010).

In the environment, Ni normally occurs at the oxidation state +2, but oxidation states 0, +1, and +3 also exist (Cempel and Nikel, 2006). Ni^2+^ (aq) ion is the dominant form throughout the pH and Eh range of most natural waters (Supplementary Figure 1). Ni forms complexes with inorganic ligands, such as halides, sulfides, sulfates, hydroxides and carbonates, and with organic oxygen, nitrogen, and sulfur compounds (Nieminen et al., 2007). Common Ni salts, e.g., chloride, nitrate and sulfate, dissolve in water, whereas carbonate and oxalate complexes are less soluble, and hydroxide and sulfide complexes are practically insoluble in water (Nieminen et al., 2007). In the environment under neutral and alkaline conditions, the mobility of Ni is considered to be low and Ni is bound on sediment or soil particles by adsorption (i.e., surface complex formation/absorption to ion exchange sites), or removed from the liquid phase by precipitation or co-precipitation (Cempel and Nikel, 2006). Under acidic conditions and in the presence of dissolved organic matter, the mobility of Ni typically increases as Ni readily forms rather stable complexes with organic ligands (Nieminen et al., 2007).

In the environment, Ni accumulates in biota and occurs inherently in food chains, and its concentration in a particular food chain may increase as a result of regional pollution (Cempel and Nikel, 2006; Nieminen et al., 2007). In low quantities, Ni is essential for humans, animals, plants and microorganisms, but high concentrations of Ni have adverse effects on biota (IARC, 2012). Although Ni is not a cumulative poison, the higher concentrations may be toxic and even carcinogenic to humans and animals (IARC, 2012). For humans and other animals, the major source of Ni exposure is oral consumption, as Ni readily accumulates to various plants. There are several species of plants, such as members of the *Brassicaceae* family (Jaffré et al., 2013), known to accumulate Ni in particularly high concentrations. Ni is also an essential nutrient for microorganisms and is known to play an important role in the biology of some bacteria, fungi and algae. Ni acts as a co-factor and participates in a variety of cellular enzymatic reactions. Several microbial enzymes have Ni as a co-factor, including urease, [NiFe]-hydrogenase, carbon monoxide dehydrogenase, acetyl-coenzyme-A decarbonylase/synthase, superoxide dismutases, and glyoxylases (Mobley and Hausinger, 1989; Hausinger, 1994; Mulrooney and Hausinger, 2003; Sukdeo et al., 2004).

Many microbes have an ability to bind Ni and can have various Ni uptake mechanisms, including biosorption on cell surfaces (based on ion-exchange, complexation, and physical adsorption), active intracellular accumulation and/or extracellular precipitation (White et al., 1995; Ledin and Pedersen, 1996; López et al., 2000; Sar et al., 2001; Mulrooney and Hausinger, 2003). Microbial (e.g., bacterial) cell walls have several potentially metal-binding functional groups, such as carboxyl, hydroxyl, sulfate, phosphate, and amine groups (White et al., 1995; Ledin and Pedersen, 1996; López et al., 2000; Sar et al., 2001). In addition, especially under low metal loading conditions sulfhydryl groups (-SH) may also be important for trace metal adsorption (Yu et al., 2014). The efficiency of Ni biosorption is dependent on various cell external factors, such as solution pH, ion activity (including competing ions), organic material contents (including complexing agents), temperature and the content of cell metabolic products in the extracellular solution (which may cause metal precipitation) (Tsezos et al., 1996; Veglió and Beolchini, 1997; Veglió et al., 1997; López et al., 2000).

For example, *Pseudomonas aeruginosa* has been reported to bind Ni, and cell wall phosphoryl, carboxyl or carbonyl groups were suggested as the major groups participating in Ni and other cation uptake in this bacterium (Sar et al., 2001). Ni uptake has also been reported in *Ps. fluorescens* 4F39 (López et al., 2000). However, in this bacterium uptake was assumed through ion exchange and precipitation on cell surfaces (López et al., 2000). In addition, Ni biosorption has also been reported, e.g., by the bacteria *Arthrobacter* sp. (Veglió et al., 1997); *Pseudomonas* sp. BP 7/26 and BP 7/15, *Alcaligenes eutrophus* CH34 and ER 121, *Ps. mendocina* AS 302, *Methylobacillus* sp. MB 127 (Tsezos et al., 1995) and the fungus *Rhizopus arrhizus* (Fourest and Roux, 1992).

In addition to straight biosorption/precipitation on cell walls, many microbes are capable of accumulating Ni inside the cell, and so far, two distinct uptake mechanisms have been identified, i.e., ATP-binding cassette (ABC)-type transporter systems and Ni-specific permeases (membrane transport proteins) (Eitinger and Mandrand-Berthelot, 2000). The first and best described ABC-type transporter for Ni was reported in *Escherichia coli* (Navarro et al., 1993; Mulrooney and Hausinger, 2003; Cherrier et al., 2005; Schalk et al., 2011; Chivers, 2015). Other bacteria, such as *Brucella suis* (Jubier-Maurin et al., 2001), *Actinobacillus pleuropneumoniae* (Bossé et al., 2001), *Helicobacter pylori* (Hendricks and Mobley, 1997), *Yersinia pseudotuberculosis, Y. pestis*, and *Y. enterocolitica* (Sebbane et al., 2002), have also been found to contain corresponding Ni-specific ABC-type transporter systems (Mulrooney and Hausinger, 2003). Ni-specific permeases have been identified, e.g., in *Ralstonia eutropha* (Eberz et al., 1989; Eitinger and Friedrich, 1991; Wolfram et al., 1995), *Bradyrhizobium japonicum* (Fu et al., 1994), *Bacillus* sp. (Maeda et al., 1994), and the yeast *Schizosaccharomyces pombe* (Eitinger et al., 2000).

In *Helicobacter pylori*, the Ni accumulation pathway involves a TonB-dependent transporter (TBDT) FrpB4 and a permease NixA (Schauer et al., 2007; Schalk et al., 2011). TBDT FrbB4 is used for Ni uptake through the outer-membrane (Schauer et al., 2007; Schalk et al., 2011). In the periplasmic space, Ni may bind to a specific protein and passes the cytoplasmic membrane by the NixA permease or another metal transporter leading to increased urease activity (Schauer et al., 2007; Schalk et al., 2011). In another *Helicobacter* species, *H. mustelae*, two distinct Ni pathways have been proposed Stoof et al. (2010) and Schalk et al. (2011). In the first one, Ni accumulation involves a TBDT NikH and a permease NixA through the outer- and inner-membrane, respectively (Stoof et al., 2010; Schalk et al., 2011). For the second, only the inner membrane ABC transporter (FecDE-CeuE) has been identified and the mechanism involved in Ni uptake across the outer membrane is unknown (Stoof et al., 2010; Schalk et al., 2011). In addition, non-specific Ni accumulation in the cytoplasm has been reported (Snavely et al., 1989, 1991; Smith and Maguire, 1995). For example, Smith and Maguire (1995) identified a CorA Mg^2+^ transporter system (metal inorganic transport) in several Gram negative bacteria. It has been suggested that CorA also mediates uptake of Ni^2+^ and Co^2+^ in addition to Mg^2+^ uptake (Snavely et al., 1989, 1991).

Significant environmental Ni deposits are found in the boreal and arctic regions (Mudd, 2010), including the Talvivaara Ni-Zn-Cu-Co mine area deposit, located in the Sotkamo area, Finland. This deposit comprises one of the largest known Ni resources in Europe (Loukola-Ruskeeniemi and Lahtinen, 2013). Large quantities of mine process waters and solid waste materials (e.g., waste rock, leached ore), having high potential for adverse environmental impacts, especially in the surrounding water systems, are found in the mining areas (Pöyry Finland Oy, 2017). A particular problem is the increased surface area of the waste rock exposed to the circulating waters, resulting in the dissolution of minerals and the leaching of toxic heavy metals like Ni. In addition, the sulfide minerals often present on mine areas are sources for acid mine drainage (AMD), which may then further dissolve potentially-toxic metals from surrounding materials and cause contamination of surrounding water resources (Akcil and Koldas, 2006). The aim of the present work was to study the interaction of Ni with newly isolated, previously unidentified bacterial strains isolated from boreal environment and to estimate their potential in the biopurification of Ni especially in the northern environment. In the northern, boreal regions, ombrotrophic bogs represent unique ecological biotopes, with distinct microbial populations. However, so far there is only limited knowledge about the heavy metal metabolism of the microbes inhabiting these northern environments. The bacterial strains newly isolated in this research, are adapted to the northern climate and environment. Thus, these bacteria have high potential for site-specific biopurification of Ni especially in the heavy-metal contaminated mine areas found in the boreal region and in the present study this potential was assessed in enhanced and controlled environment.

## Materials and Methods

### Description of the Sampling Site

In Finland, spent nuclear fuel will be stored of in deep bedrock disposal repository located on Olkiluoto Island on the western coast of Finland (Posiva, 2012). As a result of post-glacial land uplift, new bogs will be form on the repository area during the next 6000 years (Haapanen et al., 2013). At the same time period, the first possible emissions from the repository may be release to the surface ecosystem based on the biosphere safety assessments (Posiva, 2012). The sampling site, Lastensuo bog, is located about 25 km west from the Olkiluoto Island. This area has been used as a reference site for the spent nuclear fuel disposal research, representing boreal ombrotrophic bog (e.g., Mäkilä and Grundström, 2008; Lusa et al., 2015a,b,c, 2016a,b). Lastensuo is a raised bog/hummock–hollow pine bog (Mäkilä and Grundström, 2008), with an area of 4.4 km^2^ and the average depth of 6.3 m (Mäkilä and Grundström, 2008). The catchment area is till forest and the edges of the mire have been ditched. The middle part of the mire is near-treeless. Vegetation in the mire includes, e.g., slender sedge and fewflower sedge (Mäkilä and Grundström, 2008). The two main peat types are sphagnum peat and carex peat. The average degree of decomposition (von Post’s scale) is 3.4, and the annual peat growth rate is 1.1 mm/y (Mäkilä and Grundström, 2008). The bottom of the mire is mainly clay and sand. Gyttja occurs in the middle part of mire on the top of clay subsoil (Mäkilä and Grundström, 2008). The bog layers can be divided in to moss (0 m), peat (0.5–5.0 m), gyttja (5.5–6.0 m), and clay (6.7–7.0 m) (Lusa et al., 2015b). The pH of the bog profile increases with bog layer depth and the pH values vary from 3.1 (surface moss) to 5.3 (clay) (Lusa, 2017). Correspondingly the highest total Ni concentrations [15 mg/kg DW (dry weight)] are found in the gyttja layer (Lusa, 2017). High Ni concentrations (7.7 mg/kg DW) are in addition found in the moss layer. In the peat layers, significantly lower, < 2.3 mg/kg DW, Ni concentrations are found. Major cation (Na, K, Mg, Ca) concentrations are between 113 and 5178 mg/kg DW, with highest concentrations measured for Ca and lowest for Na (Lusa, 2017). The average salt concertation calculated as a sum of Na, K, Mg, and Ca is approximately 3224 mg/kg DW. In the pore water lower concentrations between 0.47 and 3.91 mg/L are found (Lusa, 2017).

### Sampling

The samples were collected from the middle part of Lastensuo bog in early summer, 2^nd^ of June, 2015. The sample plot was located on the treeless part of the bog with two small pools near the sample area. The samples for the isolation of bacteria were taken from the surface *Sphagnum* moss and from the lowest part of the bog peat layer (4.5–5.0 m). Samples were collected into 50 mL sterile centrifuge tubes using sterile tools and aseptic working methods. Peat samples were retrieved using a Russian peat corer with a nest length of 50 cm and diameter of 15 cm. The sample collection tubes were sealed with Parafilm, transported to the laboratory in cooling boxes, stored frozen at −18°C and thawed immediately before use.

The temperature of each bog layer was recorded immediately after the core sample was taken to the surface in June 2015. The temperature of the surface layer was + 10.3°C. In the lower layers, the temperature was relatively constant, with an average of + 6.6°C. In May 2015 the average temperature at the west-coastal region of Finland (Pori region) was approximately + 9°C and in June + 12°C in 2015 (Finnish Meteorological Institute [FMI], 2015). Typical average temperature in this region in July is around + 16–20°C (Finnish Meteorological Institute [FMI], 2015).

### Isolation of Bacteria

Six bacterial strains were isolated from the moss (surface) and peat (4.5–5.0 m) samples by mixing two grams of thawed, wet sample with 0.01 mol/L sterile phosphate buffer (pH 7), where after the samples were incubated aerobically for 24 h at room temperature in the dark. The resulting suspension was serially diluted in 10-fold steps to a dilution of up to 10^–5^. Aliquots of 100 μL from dilutions 10^–3^ and 10^–5^ were spread on Tryptone Soya Agar (TSA, Merckoplate) (15% Casein peptone, 5% Soya peptone, 5% NaCl; pH 7.3), R2A Agar (R2A, Merckoplate) (0.5% Proteose peptone, 0.5% Casein peptone, 0.5% Yeast extract, 0.5% Glucose, 0.5% Soluble starch, 0.3% Sodium pyruvate, 0.3% K_2_HPO_4_, 0.024% MgSO_4_; pH 7.2), Potato Dextrose Agar (PDA, Merckoplate) (4% Potato peptone, 20% Glucose; pH 5.6) and Plate Count Agar (PCA, Merckoplate) (5% Casein peptone, 2.5% Yeast Extract, 1% Glucose; pH 7.0). The plates were further incubated aerobically at 20°C for 1 week in the dark. After the incubation, pure cultures were prepared from isolated colonies by consecutive re-cultivation on corresponding agar plates.

Bacterial cells from each pure culture were Gram stained and examined using a light microscope (Nikon ECLIPSE E200) with 1000-fold magnification. Catalase activity was tested using 3% hydrogen peroxide. When bubbles were formed, the bacterium was determined positive for catalase. Oxidase activity was tested using 1% Kovács oxidase reagent. In addition, RapID ONE and RapID NF Plus (Remel) systems, and ERIC electronic code were used according to the manufacturer’s instructions for characterizing the utilization of substrates by the isolated bacterial strains.

### 16S rRNA Gene Sequencing and Phylogenetic Analysis

For the identification of bacterial isolates by sequencing, one colony of bacterial biomass was collected directly from the growth plate of each isolate using a sterile loop. The bacterial mass was suspended in the lysis buffer SL1 and Enhancer solution SE of the NucleoSpin Soil DNA extraction kit (Macherey-Nagel), after which the DNA was extracted according to the manufacturer’s instructions. The DNA was subsequently sent for PCR amplification, fragment purification, and sequencing to Macrogen, Inc., Netherlands, where PCR amplification and sequencing was conducted in both directions using primers 27f, 1492r, 518f, and 800r according to Macrogen’s protocol. Sequences were imported into Geneious Pro v. 10.1.3 (Biomatters, Ltd., Auckland, New Zealand) and processed for phylogenetic analyses. The sequences were assembled and edited by hand. In order to identify the isolated bacteria, the obtained 16S rRNA gene sequences were compared to the sequences deposited in GenBank using the blast tool in Geneious Pro. The closest matching sequences as well as relevant reference sequences were aligned using the ClustalW (Thompson et al., 1994) alignment tool in Geneious Pro. A phylogenetic tree was calculated from the alignments using the Geneious Tree Builder tool Jukes and Cantor (1969) substitution model and the topology of the tree was tested by bootstrap analyses of 1000 random resampling. The sequences have been deposited in GenBank under accession numbers MK290398-MK290403.

### Microbial Culture Conditions and Performance of Biosorption Experiments

#### Batch Experiments

Six isolated bacterial strains, *Paenibacillus* sp. IV-0-L, KV-0-YR and VV-0-L, *Methylobacterium* sp. P4-5-LR, *Paraburkholderia* sp. RP-0-BL and *Pseudomonas* sp. V4-5-SB, were selected for the Ni uptake experiments. *Paenibacillus* sp. IV-0-L, KV-0-YR and VV-0-L, and *Paraburkholderia* sp. RP-0-BL were isolated from surface moss layer and *Methylobacterium* sp. P4-5-LR, and *Pseudomonas* sp. V4-5-SB from 4.5 to 5.0 m peat layer. In addition, *Pseudomonas* sp. PS-0-L and T5-6-I (Lusa et al., 2016a, b), previously isolated from the surface moss (PS-0-L) and gyttja layer (T5-6-I) of the Lastensuo bog, were used. All bacteria were able to grow on PCA growth plates (PCA, Merckoplate, pH 7), which were selected for following stock cultures to standardize the experiments. Stocks were cultured aerobically on sterile PCA growth plates at 20°C in the dark and the colonies were transferred onto new plates weekly.

The uptake of Ni(II) by all isolated bacteria was studied using batch experiments in two different growth media. Medium A consisted of 1% Tryptone + 0.5% NaCl (pH 7) and Medium B of 1% Yeast extract + 0.5% NaCl (pH 7). These media and pH 7 were chosen for the uptake experiments as the effects of the two main components (Peptone∼Tryptone and Yeast extract) of the stock PCA (pH 7) medium was to be studied. This is because, the used bacterial strains were originally isolated on media, which all contained peptone. pH 7 was chosen, as it well corresponds to the average pH values of the natural ground waters and surface waters found in Finland, with pH between 4 and 9, highest values found in the deep ground waters (Niemi and Raateland, 2007; Posiva, 2012). The 0.5% NaCl concentration was chosen to represent to the average salt concentration of Lastensuo bog (see section “Description of the Sampling Site”). The cells were cultivated at + 4 and +20°C using incubation times of 7 and 14 days. In all uptake experiments, 14 Bq/mL of ^63^Ni(II) with a carrier concentration of 10^–10^ mol/L, corresponding to an initial Ni concentration of 6 × 10^–10^ mol/L was used. The average bacterial mass in the experiments was 0.56 g DW (dry weight)/L. Due to the weak growth of *Methylobacterium* sp. P4-5-LR a higher mass of on average 8.5 g DW/L was used. Before addition of the bacteria to the solutions, microbial suspension was prepared by adding cultivated biomass from the plates to sterile 0.01 mol/L phosphate buffer (pH 7). The mass concentration of the bacterial suspension was determined by absorbance at 600 nm.

All suspensions were incubated for 7–14 days at 4 or 20°C in the dark, after which the suspensions were centrifuged at 5000 × *g* for 15 min at room temperature. The supernatant was used for the liquid scintillation determination of ^63^Ni activity using a Tri-Carb (PerkinElmer) liquid scintillation counter. In addition, suspensions without added bacteria were prepared accordingly and measured to assure that no sorption of Ni on laboratory equipment, filters or nutrient broth solutions occurred.

The removal of Ni(II) from the solution by bacterial cells was calculated from the difference between initial and final Ni concentration in the solution and expressed as distribution coefficient (K_d_, L/kg DW): K_d_ = [(C_i_ – C_f__)_/C_f_] × (V/m), where C_i_ (Bq/L) and C_f_ (Bq/L) are the initial ^63^Ni activity and final activity concentration of the solution, V (L) is the solution volume, and m (kg DW) is the sample mass at *t* = 0.

Based on batch experiments, *Pseudomonas* sp. V4-5-SB, PS-0-L, and T5-6-I were selected for further studies due to their high Ni(II) uptake capacity.

#### The Effect of pH on Ni(II) Uptake

The effect of pH on Ni(II) uptake by *Pseudomonas* sp. V4-5-SB, PS-0-L, and T5-6-I was investigated using 0.1 mol/L NaCl at + 20^*o*^C with 7 days incubation. The 0.1 mol/L (∼5 g/L) NaCl concentration was chosen as it closely corresponds to the average salt concentration found in the Lastensuo bog from where the bacterial strains used in this study were isolated from (see section “Description of the Sampling Site”). In addition, intracellular Ni accumulation was assumed low under these scarce nutrient conditions and uptake was therefore assumed to occur principally through biosorption on bacterial cell surfaces. The pH of the samples was adjusted between 1.2 and 11.7 using 1 mol/L NaOH or HCl. This pH range corresponds to the average pH (pH 4–9) found in the natural ground and surface waters in Finland (Niemi and Raateland, 2007; Posiva, 2012). Higher pH values were not used, as at higher pH, Ni starts to precipitate as hydroxides (Nieminen et al., 2007). After 7 days incubation, the biomass was separated by centrifugation at 5000 × *g* for 15 min at room temperature and the final ^63^Ni concentration of the broth was determined using a liquid scintillation counter as descried above for batch experiments and Ni bacterial uptake was expressed as K_d_ (L/kg DW). In addition, equilibrium pH of the samples was measured.

#### The Effect of Ni(II) on Bacterial Growth

The effect of Ni(II) on bacterial growth was tested using *Pseudomonas* sp. V4-5-SB, PS-0-L, and T5-6-I with 10^–6^, 10^–3^, and 2 × 10^–2^ mol/L Ni(II) amendment. The growth of Ni(II) treated cells was compared to the cells grown without Ni(II) amendment. In these experiments, Medium A and a temperature of + 20°C was used. The cells were incubated in the dark on an orbital shaker (120 rpm) for 2 days and sampled regularly (see Figure 4) for absorbance measurement of the growth solution. The wavelength of 600 nm was used in the measurements.

#### Ni(II) Sorption Isotherms

Sorption isotherms of *Pseudomonas* sp. V4-5-SB and PS-0-L were determined in 0.1 mol/L NaCl solution buffered with 0.01 mol/L MOPS [3-(*N*-morpholino)propanesulfonicacid] or TRIS [tris(hydroxylmethyl)aminomethane] at pH 5.7 and 8.7, representing the slightly acidic surface waters and basic ground waters typically found in Finland (Niemi and Raateland, 2007; Posiva, 2012). The 0.1 mol/L (∼5 g/L) NaCl concentration was chosen to represent to the average salt concentration of Lastensuo bog (see section “Description of the Sampling Site”). The initial Ni concentration of the samples was adjusted to between 10^–10^ and 10^–3^ mol/L. Bacterial suspensions were prepared for pre-incubation by transferring bacterial colonies with a sterile loop from the growth plates into Medium A, after which the suspensions were incubated for 16 h on an orbital shaker (120 rpm) at 20°C, during which the bacterial cultures reached the plateau phase. After the incubation the absorbance of the bacterial cultures was recorded at 600 nm and 0.5 g DW/L of each bacterium was used in sorption isotherm experiments. The isotherm samples were incubated for 6 days, after which the cells were removed by centrifugation at 5000 × *g* for 15 min at the room temperature and the supernatant was used for the liquid scintillation measurement of the remaining ^63^Ni concentration and final pH of the solution. All experiments were done in triplicate. Standard samples were prepared similarly to the actual samples, except without bacterial addition. The standards were then further used to exclude the possible Ni sorption to the buffer molecules from the results. In addition, MOPS and TRIS were chosen, as Ni retention on these molecules has been previously reported non-existent or low (Perrin and Dempsey, 1974).

In order to optimize the design of sorption isotherms and to establish the most appropriate correlation, experimental isotherm data of *Pseudomonas* sp. V4-5-SB and PS-0-L were fitted to Langmuir and Freundlich isotherm models.

The linear equation for Langmuir adsorption isotherm (Langmuir, 1916) is expressed as:

(1) $$\frac{1}{q}=\frac{1}{q_{m}}+\frac{1}{q_{m}\cdot b}\cdot \frac{1}{C_{e}}$$

where q is the equilibrium concentration of metal in biomass (mg/g DW), C_e_ is the equilibrium concentration of metal in solution (mg/L), b (L/g DW), and q_m_ (mg/g DW) are Langmuir constants for forming single layer. A plot 1/q vs. 1/C_e_ should give a straight line, if the sorption process conforms to the Langmuir isotherm. Constants b and q_m_ are evaluated from the slope and intercept.

The linear equation for Freundlich adsorption isotherm (Freundlich, 1906) is expressed as:

(2) $$\log q=\log K_{F}+\frac{1}{n}\,\log C_{e}$$

where q is the equilibrium concentration of metal in biomass (mg/g DW), C_e_ is the equilibrium concentration of metal in solution (mg/L), K_f_ is a constant related to adsorption capacity and n is a constant related to adsorption intensity. If the plot log q vs. log C_e_ yields a straight line it implies that the sorption process conforms to the Freundlich isotherm. Constants K_f_ and n are evaluated from the slopes and intercepts.

The Ni uptake capacity (q, mg/g DW) was calculated from the Ni concentration of 10^–3^ mol/L according to the following equation:

(3) $$\text{q}=\text{V}\cdot \left(\frac{{\text{C}}_{\text{i}}-{\text{C}}_{\text{e}}}{\text{m}}\right)$$

where V is the sample volume (L), C_i_ is the initial metal ion concentration (mg/L), C_e_ is the equilibrium metal ion concentration (mg/L) and m is the dry weight of the biomass (g).

#### TEM and EDS Analyses

The morphology of *Pseudomonas* sp. V4-5-SB and PS-0-L incubated with 9 × 10^–4^ mol/L (∼53 mg/L) stable Ni(II) in Medium A was examined using transmission electron microscopy (TEM; Electron Microscopy Unit, Institute of Biotechnology, University of Helsinki). For these experiments, the maximum Ni concentration below lethal dose (<DL) was chosen based on the growth experiments (see section “The Effect of Ni(II) on Bacterial Growth”) to estimate the effects of Ni concentrations found in the highly contaminated mine environments. E.g., in the above mentioned Talvivaara mine area unprocessed ores and leaching heaps, extremely high Ni concentrations, around 1000 mg/kg are found (Tuovinen et al., 2018). In addition, samples without Ni amendment were prepared. The cells were incubated at + 20°C for 6 days, which after the cell mass was separated as described above for batch experiments. After centrifugation, 1 ml of 5% glutaraldehyde was added to the bacterial pellet and allowed to stand at room temperature for 2 h. Thereafter, the fixed cells were centrifuged (5000 × *g*, 15 min) and transferred to 0.01 mol/L phosphate buffer.

Cells were dehydrated through an ethanol series and embedded in Taab hard epon and polymerized at 60°C. Thin sections were cut using a Leica ultracut UCT ultramicrotome (Leica Mikrosysteme GmbH, Austria) and collected on single-slot copper grids. Section thickness of 60 nm was used for the morphological examinations. The sections, unstained and stained with uranyl acetate and lead citrate, were examined using Jeol JEM-1400 TEM under standard operating conditions.

Energy dispersive X-ray spectroscopy (EDS; Department of Chemistry, University of Helsinki) was used for the determination of elemental compositions of bacterial biomass incubated with Ni. EDS samples were cultured as described for the TEM samples above. After incubation, the cells were separated by centrifugation at 5000 × *g* for 15 min at room temperature followed by washing of the remaining bacterial pellet twice with 0.01 mol/L phosphate buffer. Thereafter, the pre-frozen bacterial pellet (at −18°C) was lyophilized using an Alpha 1-4 LSCbasic lyophilizer (Christ). The lyophilized bacterial mass was fixed to the sample stub/support with conductive carbon tape and both the sample and plug were coated with 10 nm carbon layer using Leica EM ACE200 coating system (Leica Microsystems). Qualitative elemental analysis of samples was performed using Oxford INCA 350 Energy dispersive X-ray microanalysis system at 20 kV under standard operating conditions. The detection limit of EDS is estimated to be about 0.1 wt%.

#### Fourier Transform Infrared Spectroscopy

Fourier transform infrared (FTIR) spectroscopy [Bruker ALPHA FTIR Spectrometer (Platinum ATR)] was used to study the functional groups present on the cell surfaces of *Pseudomonas* sp. strains (V4-5-SB, PS-0-L, and T5-6-I). In these experiments, lyophilized biomass was used and the samples were cultured and prepared as described above for EDS. The IR spectra were recorded from Ni(II) treated samples, as well as from samples without Ni(II) amendment.

#### Protein Expression in the Presence of Ni(II)

The stress responses generated by Ni(II) addition were examined to identify potentially important proteins involved in Ni(II) metabolism. SDS-PAGE and MALDI-TOF (Matrix-Assisted Laser Desorption/Ionization – Time of flight) – mass spectrometry were used to study the proteins expressed in *Pseudomonas* sp. strains (V4-5-SB, PS-0-L, and T5-6-I) in the presence of Ni (9 × 10^–4^ mol/L) and compared to the cells grown without Ni(II) amendment. Samples were prepared similarly to the TEM samples described above, except of a shorter incubation time of 4 days. The soluble protein fractions from control untreated and Ni(II) treated cells were isolated using the BugBuster method according to the manufacturer’s instructions.

The isolated proteins were separated with SDS-PAGE using pre-cast ClearPage SDS 10% gels with constant voltage of 175 V for 45 min. SDS-PAGE samples were prepared following the manufacturer’s instructions for ClearPage gels, using 3 μL of 10 × DTT per 13 μL of sample volume. Equal amounts of protein (ca. 3 μg) were loaded onto each well. TEO-Tricine (ClearPAGE SDS Running Buffer) was used as running buffer. The proteins were visualized using the Blue BANDIT protein stain and the visible protein lines of gel were compared to the protein standard (The ClearPAGE Two-Color SDS Marker, CBS Scientific).

Additionally, MALDI-TOF was used for the mass determination of proteins isolated from *Pseudomonas* sp. PS-0-L by using the BugBuster method. For MALDI-TOFF the samples were sent to the Proteomics Unit, Viikki, University of Helsinki, where the protein solutions were purified and concentrated using the ZipTip –method and MALDI-TOF was run in sinapic acid/acetonitrile buffer.

### Statistical Analyses

To study the statistical difference between the different growth conditions, i.e., the difference between nutrient broths A and B and the temperatures + 4 and +20°C the analysis of variance was performed using Microsoft Excel Data Analysis and one-way ANOVA at the *p* < 0.05 level. Analysis of variance was done for all studied bacteria separately for temperature and nutrient broth. In addition, the ANOVA analysis for the statistical differences in the uptake between different bacterial groups (*Pseudomonas*, *Paenibacillus, Paraburkholderia*/*Methylobacterium*) was done. Kurtosis and skewness of the data was tested to examine the normality of the data. As both skewness and kurtosis were found to differ from zero, log transformed data was used for the one-way ANOVA tests of the data.

## Results

### Characterization of the Microbial Isolates

**FIGURE 1:**
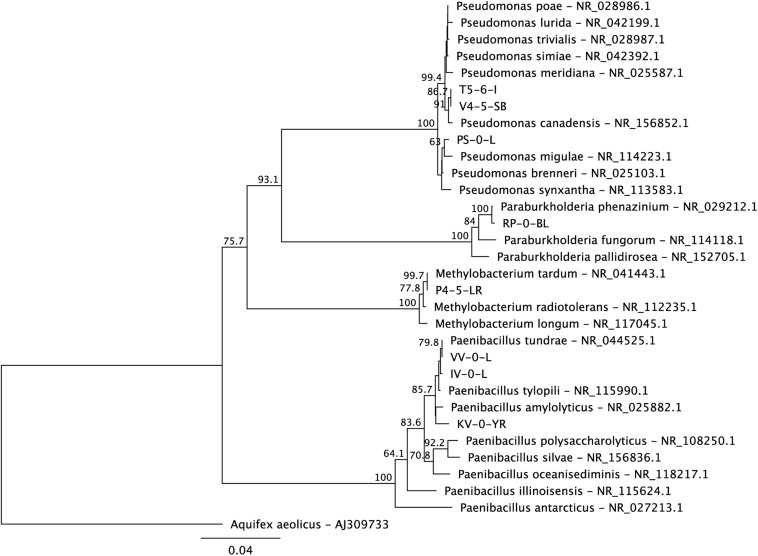
A maximum likelihood phylogenetic tree of the 16S rRNA genes of the new Lastensuo isolates in respect to the closest relative reference strains. The topology of the tree was tested by bootstrap analyses of 1000 random resamplings and nodes with > 50% bootstrap support are indicated. The scale bar represents number of nucleotide substitutions per site.

Based on the 16S rRNA gene sequences, the isolated bacteria affiliated with genera *Paenibacillus* (KV-0-YR, IV-0-L, and VV-0-L), *Methylobacterium* (P4-5-LR), *Paraburkholderia* (RP-0-BL), and *Pseudomonas* (V4-5-SB) (Figure 1). The utilization of substrates by these strains was tested using the RapID ONE and RapID NF Plus systems (Remel) (Table 1). Five of the newly isolated bacteria were stained Gram negative (*Paenibacillus* sp. IV-0-L and VV-0-L; *Methylobacterium* sp. P4-5-LR; *Paraburkholderia* sp. RP-0-BL; and *Pseudomonas* sp. V4-5-SB) and one Gram positive (*Paenibacillus* sp. KV-0-YR) (Table 1). Two of the three *Paenibacillus* (IV-0-L and VV-0-L) isolates were found Gram negative, although *Paenibacillus* strains are usually stained Gram positive. However, also previously (Grady et al., 2016) *Paenibacillus* have been shown to have mixed Gram staining, and they may also stain Gram negative.

**TABLE 1:** Biochemical characteristic of *Paenibacillus* sp. KV-0-YR, *Paenibacillus* sp. IV-0-L, *Paenibacillus* sp. VV-0-L, *Paraburkholderia* sp. RP-0-BL, *Methylobacteria* sp. P4-5-LR, and *Pseudomonas* sp. V4-5-SB.

**Strain**	**KV-0-YR**	**IV-0-L**	**VV-0-L**	**RP-0-BL**	**P4-5-LR**	**V4-5-SB**
Phylum	Firmicutes	Firmicutes	Firmicutes	Proteobacteria	Proteobacteria	Proteobacteria
Class	Bacilli	Bacilli	Bacilli	β-Proteobacteria	α-Proteobacteria	γ-Proteobacteria
Genus	*Paenibacillus*	*Paenibacillus*	*Paenibacillus*	*Paraburkholderia*	*Methylobacterium*	*Pseudomonas*

**Accession number**	**MK290399**	**MK290398**	**MK290403**	**MK290401**	**MK290400**	**MK290402**

Cell morphology	Rod	Rod	Rod	Rod	Rod	Rod
Gram	+	−	−	−	−	−
Oxidase	+	+	−	−	+	+
Catalase	+	+	+	−	+	+

**RapID panel**		**NF Plus**	**ONE**	**ONE**	**NF Plus**	**NF Plus**

Indole		−	−	−	−	−
Urea		−	+	−	+	−
Sodium nitrate		−			+	−
Glucose		−			−	−
Malonate			−	−		
**Amino acid hydrolysis:**						
Arginine		−	−	−	−	−
Lysine			−	−		
Ornithine			−	−		
**Utilization of carbohydrates:**						
Sorbitol			−	−		
Sugar aldehyde			−	+		
Adonitol			+	+		
**Hydrolysis of:**						
Aliphatic thiol		−	−	−	−	+
Fatty acid ester			−	+		
Triglyceride		+			−	+
**Enzymatic hydrolysis of glucoside or phosphoester:**						
*p*-Nitrophenyl-β,D-glucuromide			−	−		
ρ-Nitrophenyl-*N*-acetyl-β,D-glucosaminide		−	−	−	−	−
*p*-Nitrophenyl-β,D-xyloside			+	−		
ρ-Nitrophenyl-β,D-glucoside		+	+	−	−	−
ρ-Nitrophenyl-α,D-glucoside		+			+	+
ρ-Nitrophenyl-phosphoester		+			+	+
ρ-Nitrophenyl-β,D-galactoside		+			-	+
*o*-Nitrophenyl-β,D-galactoside			+	−		
**Enzymatic hydrolysis of aryl amide:**						
γ-Glutamyl-β-naphthylamide		+	−	+	+	+
Pyrrolidine-β-naphthylamide		+			−	−
Pyrrolidonyl-β-naphthylamide			+	−		
Proline-β-naphthylamide		−	−	+	+	+
Tryptophane-β-naphthylamide		+			+	+

*Remel RapID ONE and RapID NF Plus systems were used to characterize the utilization of substrates by these strains. The Remel RapID ONE system was suitable for gram negative and oxidase negative bacteria and Remel RapID NF Plus system for gram negative and oxidase positive bacteria. Empty cell means that the substrate was not tested.*

Of the *Paenibacillus* sp. strains, VV-0-L was oxidase negative and strains KV-0-YR and IV-0-L were oxidase positive. Due to the Gram negative staining of *Paenibacillus* sp. strains IV-0-L and VV-0-L, these were further characterized with RapID panels designed for Gram negative bacteria. The substrate utilization patterns of these bacteria were similar, with the exception that *Paenibacillus* sp. IV-0-L could hydrolyze γ-glutamyl-β-naphthylamide and *Paenibacillus* sp. VV-0-L could not. In turn, *Paenibacillus* VV-0-L was able to utilize nitrate, while *Paenibacillus* sp. IV-0-L was not.

*Pseudomonas* sp. V4-5-SB, PS-0-L, and T5-6-I had partially similar substrate utilization patterns. However, only *Pseudomonas* sp. V4-5-SB showed positive oxidase activity, while *Pseudomonas* sp. PS-0-L and T5-6-I (Lusa et al., 2016a) were oxidase negative. None of the tested *Pseudomonas* sp. strains (V4-5-SB, PS-0-L, and T5-6-I) were able to utilize glucose. In addition, *Pseudomonas* sp. V4-5-SB was unable to utilize nitrate while *Pseudomonas* sp. PS-0-L and T5-6-I used nitrate (Lusa et al., 2016a). All *Pseudomonas* sp. strains hydrolyzed triglyceride, but the hydrolysis of aliphatic thiol was variable. Overall, *Pseudomonas* sp. PS-0-L appears to be more similar to *Pseudomonas* sp. T5-6-I, and *Pseudomonas* sp. V4-5-SB differed from these two strains in the utilization of substrates. The results of the 16S rRNA gene sequences, Gram staining, catalase and oxidase activity tests and RapID systems for *Pseudomonas* sp. PS-0-L and T5-6-I have been previously reported by Lusa et al. (2016a).

### Uptake of Ni(II) by *Paenibacillus* sp., *Methylobacterium* sp., *Paraburkholderia* sp., and *Pseudomonas* sp. Strains

**FIGURE 2:**
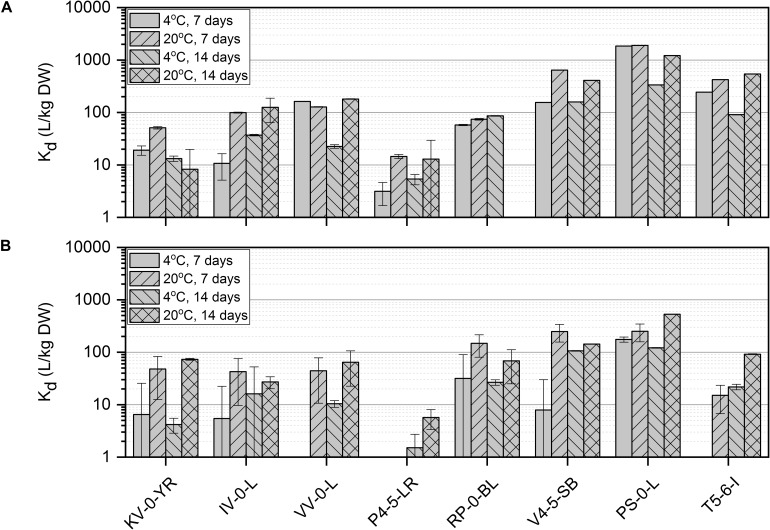
Ni(II) uptake (initial Ni(II) concentration 6 × 10^–10^ mol/L) by isolated *Paenibacillus* sp. KV-0-YR, IV-0-L, and VV-0-L; *Methylobacterium* sp. P4-5-LR; *Paraburkholderia* sp. RP-0-BL; and *Pseudomonas* sp. V4-5-SB, PS-0-L, and T5-6-I in Media A **(A)** and B **(B)** at +4 and +20°C with 7 and 14 days incubation time. Values represent the geometric means of triplicate trials and uncertainty bars are the standard error of the geometric mean.

The effect of temperature, incubation time, nutrient conditions, and pH on Ni(II) removal by the isolated bacteria was studied using batch experiments. Since there is only limited knowledge about the metabolism of the bacteria inhabiting the northern bogs and because the ability of these bacteria to interact with Ni is not known, batch experiments were conducted in an enhanced and controlled environment. All studied bacterial strains removed Ni(II) from the solution and the uptake efficiency was affected by nutrient source, incubation time and temperature (Figure 2). The highest Ni uptake was shown by *Pseudomonas* sp. PS-0-L in Medium A at + 20°C after 7 days incubation. Compared to *Pseudomonas* sp. V4-5-SB (max K_d_ 640 L/kg DW) and T5-6-I (max K_d_ 420 L/kg DW), Ni uptake was three- to five-fold higher in *Pseudomonas* sp. PS-0-L. In PS-0-L, a maximum K_d_ value of 1890 L/kg DW was recorded and this strain showed higher Ni removal compared to the other two *Pseudomonas* sp. strains irrespective of the used growth media, temperature or incubation time. In all *Pseudomonas* sp. strains, the average Ni uptake was observed to increase with increasing incubation temperature from + 4 to + 20°C. In addition, longer incubation time (14 days, compared to 7 days) decreased Ni(II) removal in Medium A for all studied strains. Only *Pseudomonas* sp. T5-6-I removed Ni(II) more efficiently after 14 days incubation, compared to the 7 days incubation at + 20°C. Contrary to Medium A, in Medium B incubation time and temperature had no clear effect on Ni(II) uptake by any of the studied *Pseudomonas* sp. strains. In Medium B, the highest Ni(II) uptake was shown by *Pseudomonas* sp. PS-0-L (max K_d_ 529 L/kg DW) and T5-6-I (max K_d_ 92 L/kg DW) at + 20°C using 14 days incubation time. For *Pseudomonas* sp. V4-5-SB the maximum K_d_ value of 248 L/kg DW was observed after 7 days incubation.

The highest uptake by the studied *Paenibacillus* sp. strains (IV-0-L and VV-0-L) was observed in Medium A, with maximum K_d_ values of 125 L/kg DW (+ 20°C, 14 days) and 180 L/kg DW (+ 20°C, 14 days) for IV-0-L and VV-0-L, respectively. For *Paenibacillus* sp. KV-0-YR, the highest uptake (max K_d_ 73 L/kg DW at + 20°C, 14 days) was observed in Medium B.

For *Paraburkholderia* sp. RP-0-BL the maximum K_d_ of 148 L/kg DW was observed at + 20°C after 7 days incubation in Medium B. For *Methylobacterium* sp. P4-5-LR only low Ni(II) uptake was observed, with a maximum uptake of 15 L/kg DW at + 20°C after 7 days incubation in Medium A. Compared to the other studied bacterial strains, *Methylobacterium* sp. P4-5-LR showed significantly lower Ni(II) retention regardless of the growth medium, temperature, or incubation time used than any of the other strains. In Medium B, Ni(II) uptake by *Methylobacterium* sp. P4-5-LR was non-existent, with recorded K_d_ values between 2 and 6 L/kg DW.

**FIGURE 3:**
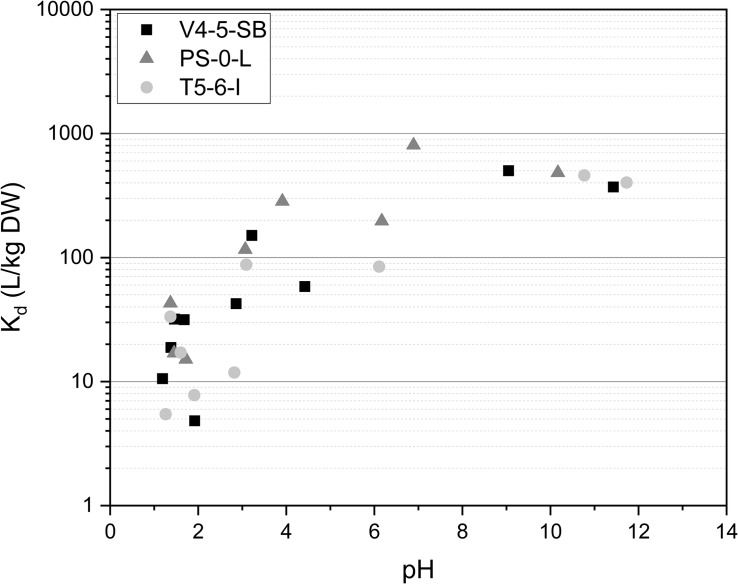
The effect of pH on Ni uptake by *Pseudomonas* sp. V4-5-SB, PS-0-L, and T5-6-I in 0.1 mol/L NaCl at pH-range 1.2–11.7. Based on pH-Eh diagram (Supplementary Figure 1) Ni is found as hydroxide above pH 10.

The effect of pH on Ni(II) uptake by *Pseudomonas* sp. V4-5-SB, PS-0-L, and T5-6-I was in addition investigated at the pH-range of 1.2–11.7. In all studied *Pseudomonas* sp. strains a strong effect of pH on Ni(II) adsorption was observed (Figure 3) and Ni uptake increased with the increasing pH. The maximum K_d_ values were 502, 807, and 461 L/kg DW for V4-5-SB, PS-0-L, and T5-6-I, respectively. The highest Ni uptake was observed at pH 6–9 depending on strain. As mentioned above, this pH range well corresponds to the average pH values of the natural ground and surface waters found in Finland (Niemi and Raateland, 2007; Posiva, 2012).

### Bacterial Growth Under Ni(II) Stress

**FIGURE 4:**
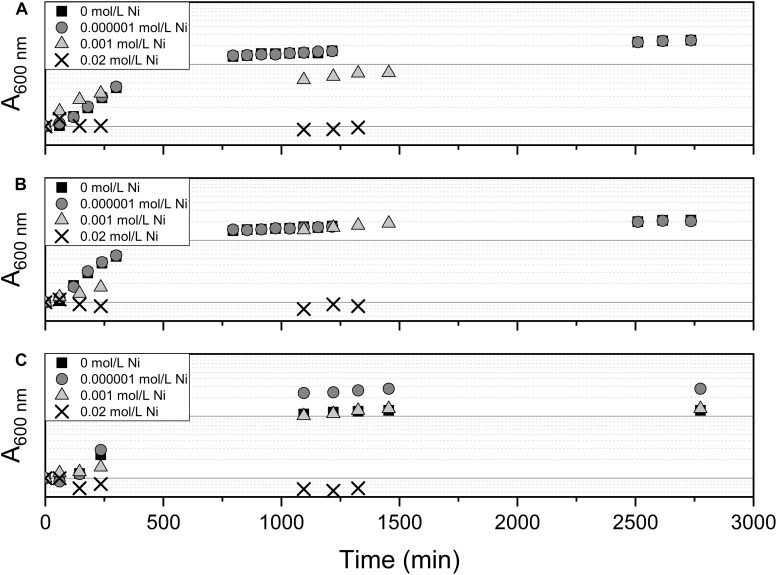
The effect of Ni on bacterial growth by *Pseudomonas* sp. V4-5-SB **(A)**, PS-0-L **(B)**, and T5-6-I **(C)** during the 2-day growth period in Medium A. Tested Ni concentrations were 0, 0.000001, 0.001, and 0.02 mol/L.

The effect of 10^–6^, 10^–3^, and 2 × 10^–2^ mol/L concentration of Ni(II) on bacterial growth was tested with *Pseudomonas* sp. V4-5-SB, PS-0-L, and T5-6-I using a 2-day growth period and an incubation temperature of + 20°C. In these experiments, no significant differences between the growth of cells with 10^–6^ and 10^–3^ mol/L Ni(II) and cells without Ni(II) were observed (Figure 4). In both treatments, the exponential increase in cell growth was observed after a lag phase between 60 and 720 min (1–12 h). However, no bacterial growth was observed at the highest Ni(II) concentration (2 × 10^–2^ mol/L) with tested *Pseudomonas* sp. V4-5-SB, PS-0-L and T5-6-I, indicating a potential toxicity for the bacteria.

### Morphology and Elemental Composition of the Cells

**FIGURE 5:**
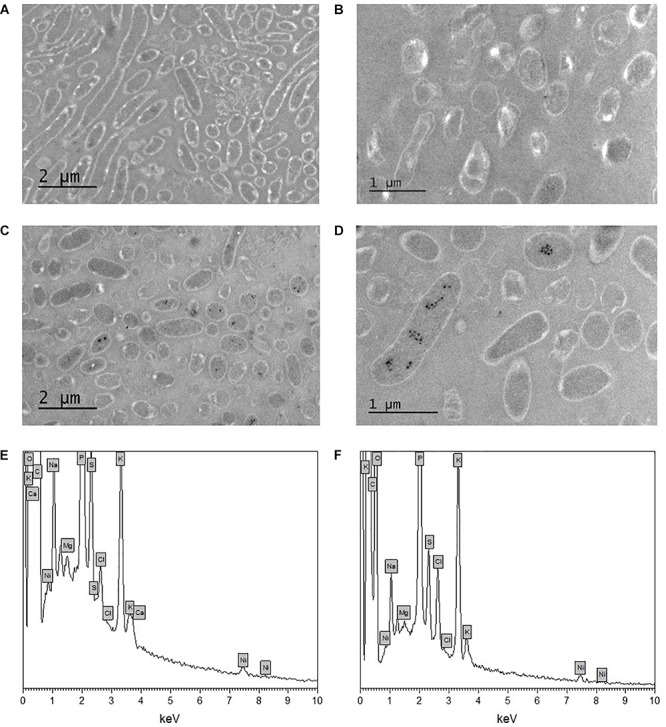
Transmission electron microscopy (TEM) images of unstained *Pseudomonas* sp. V4-5-SB **(A,C)** and PS-0-L **(B,D)** cells incubated without nickel **(A,B)** and with nickel (9 × 10^–4^ mol/L) **(C,D)**, and EDS spectra of *Pseudomonas* sp. V4-5-SB **(E)** and PS-0-L **(F)** representing the presence of nickel in bacterial biomass.

The morphology of *Pseudomonas* sp. V4-5-SB and PS-0-L Ni(II) treated cells was studied using TEM. This was done for more detailed information on Ni localization either on the bacterial cell walls or inside the cells. In the TEM images dense crystalline formations were observed inside the bacterial cells after incubation with 9 × 10^–4^ mol/L Ni(II) solution (Figures 5C,D) in both studied bacteria. In the cells grown without Ni(II) amendment, corresponding crystalline formations were not observed (Figures 5A,B). In the corresponding EDS spectra (Figures 5E,F), the K_α_ and L_α_ X-ray peaks of Ni are seen at 7.5 and −0.85 keV, respectively. However, the intensities of Ni X-ray peaks remained relatively low in the measured spectra. In addition to Ni, other elements detected by EDS were carbon, oxygen, phosphorus, potassium, sulfur, chlorine, sodium, magnesium, and calcium. Two major elements, potassium and phosphorus, are probably from the pre-treatment step of the samples and from the phosphate buffer used. Other elements are naturally present on the bacterial surfaces.

### The Effect of Ni(II) on Protein Expression

**FIGURE 6:**
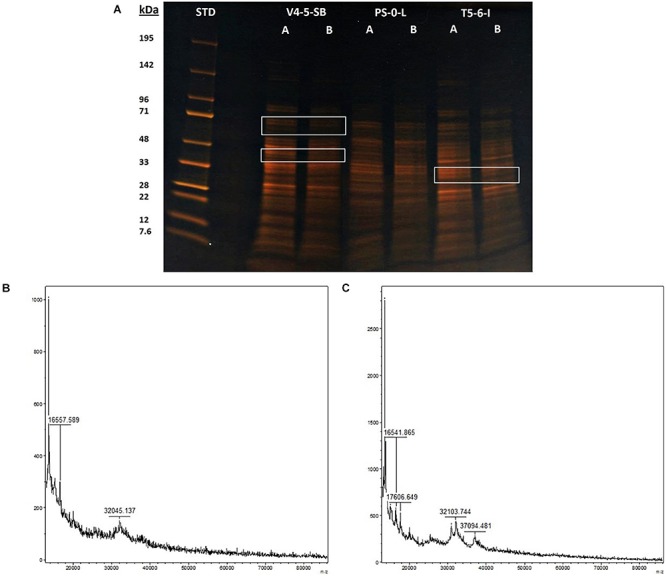
Protein profiles resolved on a 10% acryl amide SDS-PAGE gel, lanes marked with **(A)** and **(B)** indicate bacterial cells incubated without Ni **(A)** and with Ni **(B)**. Equal amounts of protein (ca. 3 μg) were loaded onto each well. MALDI-TOF spectra of PS-0-L **(B)** control and **(C)** Ni treated cells.

The stress responses of *Pseudomonas* sp. V4-5-SB, PS-0-L, and T5-6-I to the presence of Ni(II) were investigated using SDS-PAGE (V4-5-SB, PS-0-L, and T5-6-I) and MALDI-TOFF (PS-0-L). In the SDS-PAGE experiments, no major differences were observed in the expressed protein profiles by *Pseudomonas* sp. V4-5-SB, PS-0-L, and T5-6-I induced by Ni(II) amendment, compared to the samples incubated without Ni(II) (Figure 6A). However, the intensities (corresponding to the concentrations) of the expressed proteins were clearly lower at the approximate regions of ∼60–71 and ∼40–45 kDa in *Pseudomonas* sp. V4-5-SB induced by the presence of Ni(II). A slight decrease in the range of ∼32 kDa in the line intensities was also observed in *Pseudomonas* sp. T5-6-I incubated with Ni(II), compared to the cells without Ni(II) (Figure 6A). As the resolution of the SDS-PAGE is relatively low, when using crude extracts with several proteins present and especially on the low kDa proteins, MALDI-TOFF was used for better resolution of proteins with low molecular mass. In the MALDI-TOFF spectra (Figures 6B,C), several additional protein peaks were observed at the approximate region of 13–40 kDa in PS-0-L incubated with Ni(II) (Figure 6C), compared to the sample without Ni(II) (Figure 6B).

### Ni(II) Sorption Isotherms

**FIGURE 7:**
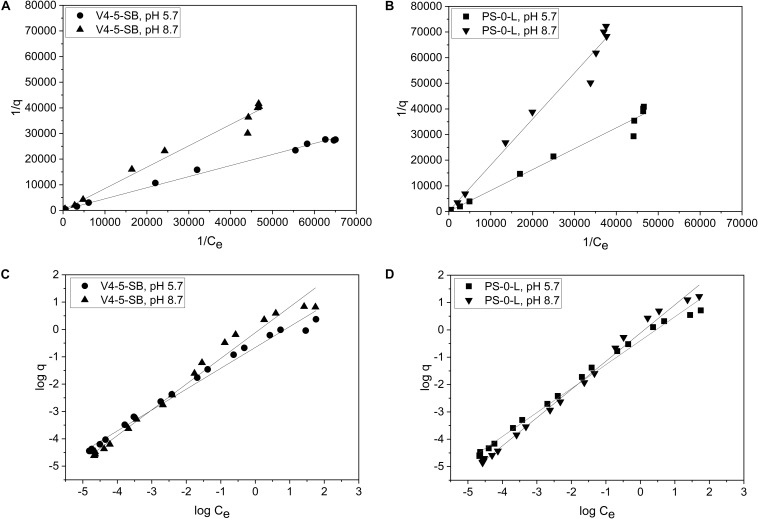
The biosorption isotherms of *Pseudomonas* sp. V4-5-SB **(A,C)** and PS-0-L **(B,D)** at pH 5.7 and 8.7. Values represent the geometric means of triplicate trials and the uncertainty bars are the standard error of the geometric mean. Graphs **(A,B)** present the plot 1/q vs. 1/C_e_ and a straight line implies that the sorption process conforms to the Langmuir isotherm. Graphs **(C,D)** present the plot log q vs. log C_e_ and a straight line implies that the sorption process conforms to the Freundlich isotherm.

Due to the high Ni(II) uptake observed in the batch K_d_ experiments, *Pseudomonas* sp. V4-5-SB and PS-0-L were used in the following sorption isotherm experiments. Both Langmuir and Freundlich isotherm models were tested and the Ni uptake by the tested bacterial strains were found to follow both equations (Figure 7). The Langmuir model gave a good fit for the Ni(II) uptake for both strains at pH 5.7 and 8.7 with the *R*^2^ values of 0.997 and 0.986, and 0.987 and 0.989, for V4-5-SB and PS-0-L at pH 5.7 and 8.7, respectively. At pH 5.7 and 8.7, the q_m_ values of *Pseudomonas* sp. V4-5-SB were 0.00494 and 0.00496 mg/g DW, and the *b* values were 468 and 242 L/g DW (Table 2), respectively. For *Pseudomonas* sp. PS-0-L, the q_m_ values were 0.0265 and 0.00756 mg/g DW, and the *b* values were 46.3 and 73.6 L/g DW at pH 5.7 and 8.7, respectively.

**TABLE 2:** Constants q_m_ and *b* evaluated from the intercepts and slopes of graphs A and B in Figure 7.

		***Langmuir***			***Freundlich***		
***Pseudomonas* sp. strain**	***pH***	***q*_*m*_**	***b***	***R*^2^**	**$\frac{1}{n}$**	***K*_*F*_^∗^**	***R*^2^**
V4-5-SB	5.7	0.00494	468	0.997	0.763	0.221	0.987
	8.7	0.00496	242	0.986	0.941	0.764	0.982
PS-0-L	5.7	0.0265	46.3	0.987	0.876	0.403	0.992
	8.7	0.00756	73.6	0.989	1.03	0.769	0.993

*Similarly, constants K_*f*_ and 1/n are evaluated from the slopes and intercepts of graphs C and D in Figure 7. ^∗^Unit of q_*m*_ is mg/g DW, b is L/g DW and K_*f*_ is mg^1–1/n^L^1/n^g^–1^.*

The Freundlich isotherm model showed also a good fit for the Ni(II) uptake by *Pseudomonas* sp. V4-5-SB and PS-0-L at pH 5.7 and 8.7 with the *R*^2^ values of 0.987 and 0.982; and 0.992 and 0.993, for V4-5-SB (at pH 5.7 and 8.7) and PS-0-L, respectively. At pH 5.7, the Freundlich adsorption constant (K_f_ value) of *Pseudomonas* sp. V4-5-SB was 0.221 mg L^1/n^ g^–1^ and the 1/n value 0.763 (Table 2). Correspondingly, at pH 8.7, the K_f_ value was 0.764 mg L^1/n^ g^–1^ and the 1/n value was 0.941. For *Pseudomonas* sp. PS-0-L, K_f_ values were 0.403 mg L^1/n^ g^–1^ and 0.769 mg L^1/n^ g^–1^; and the 1/n values were 0.876 and 1.03 at pH 5.7 and 8.7, respectively.

At low initial Ni concentrations, Freundlich isotherms behaved ideally but after 10^–4^ mol/L a slight deflection in the Freundlich isotherms at both pH 5.7 and 8.7 was observed (Figures 7A,B). In addition, the equilibrium uptake capacity (q) of *Pseudomonas* sp. V4-5-SB and PS-0-L increased with increasing solution pH and initial Ni concentration. The highest *q* values were observed as the initial Ni concentration of 10^–3^ mol/L was used. The corresponding *q* values for *Pseudomonas* sp. V4-5-SB and PS-0-L were 2.4 and 5.2 mg/g DW at pH 5.7, and 6.6 and 16.8 mg/g DW at pH 8.7, respectively.

### FTIR Analysis

**FIGURE 8:**
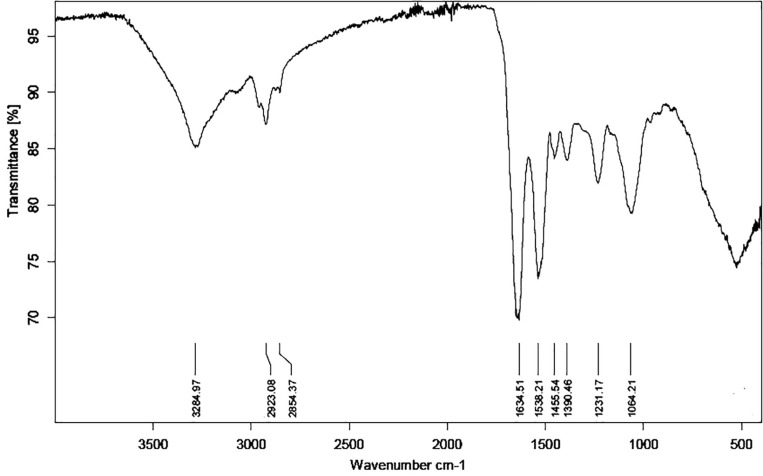
Fourier transformation infrared spectroscopy (FTIR) spectrum of *Pseudomonas* sp. PS-0-L. The spectra of *Pseudomonas* sp. V4-5-SB and T5-6-I were similar to that of PS-0-L and the presence of nickel did not change the situation (spectra are not shown).

As Ni(II) uptake conformed both Freundlich and Langmuir isotherms, FTIR analysis was performed to obtain more information on the functional groups present on the bacterial cell surfaces and possibly participating Ni(II) uptake in the *Pseudomonas* sp. strains V4-5-SB, PS-0-L, and T5-6-I. In these experiments, no significant differences were observed in the functional groups of different *Pseudomonas* sp. strains, and alcoholic and carboxylic hydroxyl, amine, alkane and phosphate groups were observed in all bacteria (Figure 8 and Table 3). In addition, nickel was not found to affect the presence of functional groups on the cell surface. All bacteria showed absorption peaks around 3300 cm^–1^, which indicate O-H and N-H stretching and shows the presence of alcoholic hydroxyl groups and amine groups in these bacteria. In addition, the bands at 1500 and 1200 cm^–1^ can be assigned to the N-H stretch and C-N stretch caused by amine groups. In addition, in all cells, a band at about 1000–1100 cm^–1^ was observed, representing a P = O stretch caused by a phosphate group. The overall spectral analysis supports the potential role of alcoholic hydroxyl, carboxylic carbonyl, and amine groups in Ni(II) binding in the *Pseudomonas* sp. V4-5-SB, PS-0-L, and T5-6-I strains.

**TABLE 3:** Major absorption peaks observed in the FTIR spectra of *Pseudomonas* sp. V4-5-SB, PS-0-L and T5-6-I (incubated without and with Ni addition) and possible functional groups involved in Ni binding on cell surface.

**V4-5-SB**		**PS-0-L**		**T5-6-I**		**Absorption bands from literature (cm**^–^**^1^)^∗^**	**Functional group**
**Control cells (cm**^–^**^1^)**	**Ni treated cells (cm**^–^**^1^)**	**Control cells (cm**^–^**^1^)**	**Ni treated cells (cm**^–^**^1^)**	**Control cells (cm**^–^**^1^)**	**Ni treated cells (cm**^–^**^1^)**		
3276	3277	3285	3277	3274	3287	3400–3650 3300–3500	O-H alcohol N-H amine
2921	2927	2923	2926	2925	2928	2850–2960 2500–3100	C-H alkane O-H carboxylic acid
2853	2856	2854	2852	2854	2855		
1635	1637	1635	1652	1646	1635	1670–1780 1620–1680	C = O carboxylic acid C = C alkane
1538	1534	1538	1538	1540	1538	1550–1640	N-H amine
1456	1450	1456	1456	1456	1456	1350–1480	C-H alkane
1387	1388	1390	1386	1389	1386		
1234	1234	1231	1234	1234	1234	1030–1230	C-N amine
1069	1066	1064	1059	1069	1065	1000–1100	P = O phosphate

*^∗^Hase, 1992; McMurry, 2008.*

### Statistical Analyses

Based on the analysis of variance (ANOVA), the different growth conditions (i.e., nutrient broth and temperature) affected the Ni uptake. The differences in the K_d_ values in Media A and B, and at temperatures + 4 and + 20°C were found statistically significant at the *p* < 0.05 level (Table 4). In addition, the difference in the Ni uptake between different bacterial groups [i.e., *Pseudomonas, Paenibacillus, Paraburkholderia/Methylobacterium* (other bacteria)] were statistically significant at 0.05 level (Table 4).

**TABLE 4:** Statistically significant differences in the Ni uptake between the different bacterial strains analyzed by ANOVA.

	**I**			**II**			**III**		
	***F***	**F_crit_**	***p*-Value**	***F***	**F_crit_**	***p*-Value**	***F***	**F_crit_**	***p*-Value**
KV-0-YR	0.021	4.6	0.89	4.5	4.6	0.052	45	3.1	1.3×10^−15^
IV-0-L	2.6	4.6	0.13	5.9	4.6	0.029			
VV-0-L	5.1	4.7	0.043	3.5	4.7	0.084			
P4-5-LR	7.5	4.6	0.016	8.2	4.6	0.012			
RP-0-BL	1.1	4.8	0.33	1.4	4.8	0.26			
V4-5-SB	6.7	4.5	0.020	8.2	4.5	0.011			
PS-0-L	11	4.4	0.0041	1.8	4.4	0.19			
T5-6-I	17	4.4	0.00065	0.20	4.4	0.66			

*I = difference in the Ni uptake between medium A compared to medium B, when all bacterial isolates were combined. II = difference in the Ni uptake at +4°C compared to Ni uptake at +20°C, when all bacterial isolates were combined. III = difference in the Ni uptake between bacterial groups, when *Pseudomonas*, *Paenibacillus*, and *Paraburkholderia/Methylobacterium* (other bacteria) were combined. The underlined results are statistically significant at 0.05 level.*

## Discussion

Ni mining and other industrial, as well as domestic use can cause increased, potentially toxic emissions of Ni to the environment. In addition, nuclear energy production results in long-lived, possibly highly mobile radioactive isotopes of Ni, ^59^Ni and ^63^Ni, potentially harmful for the biosphere. Significant environmental Ni deposits are found, e.g., in North America and Russia (Mudd, 2010), in the boreal and arctic regions with significant portions of the world’s mires, bogs, and other peatlands (Joosten and Clarke, 2002). In the boreal region, ombrotrophic bogs represent ecological biotopes, with distinct microbial populations, but so far, only limited knowledge is available about the metabolism of the microorganisms inhabiting these areas. Many environmental bacteria can bind Ni and other heavy metals and two types of uptake processes, biosorption and bioaccumulation, have been presented (e.g., Wang and Chen, 2009). In biosorption, the functional groups of the bacterial cell wall, such as hydroxyl, carboxyl and phosphate groups, participate in metal binding either by adsorption, ion-exchange, precipitation, or complexation (White et al., 1995; Ledin and Pedersen, 1996; López et al., 2000; Sar et al., 2001). In contrast to biosorption, in bioaccumulation, metal is transported through the bacterial cell wall and membrane and the transport is typically linked to the cell metabolism and is consequently a substantially slower process than direct biosorption on to cell walls. In addition to the characteristics of a specific bacterial strain, the efficiency of metal uptake is dependent on various external factors, such as pH, competing ions, organic material, cell metabolism and temperature, because the adsorption reaction can change as a function of changing conditions (Tsezos et al., 1996; Veglió and Beolchini, 1997; Veglió et al., 1997; López et al., 2000; Jiang et al., 2004).

In the present study, new bacterial strains were isolated from the acidic, nutrient-poor, boreal Lastensuo bog. The isolated strains belonged to the genera *Paenibacillus* (KV-0-YR, IV-0-L, and VV-0-L), *Methylobacterium* (P4-5-LR), *Paraburkholderia* (RP-0-BL), and *Pseudomonas* (V4-5-SB). Previously, we characterized the bacterial community of this bog by 454 pyrosequencing and found 40 different bacterial phyla in the peat profiles from the surface to the 6 m depth (Tsitko et al., 2014). *Acidobacteria* and α*-* and γ*-Proteobacteria* dominated the surface community. In addition, *Chloroflexi, Bacteroidetes, Verrucomicrobia, Planctomycetes*, and *Spirochaeta* formed the majority of the bacterial phyla present in this bog. These bacteria were, however, not captured in pure cultures in our study.

In the current work, the uptake of Ni by *Paenibacillus* (KV-0-YR, IV-0-L, and VV-0-L), *Methylobacterium* (P4-5-LR), *Paraburkholderia* (RP-0-BL), and *Pseudomonas* (V4-5-SB) was studied using batch experiments under favorable growth conditions for the isolated bacteria. Ni uptake varied substantially between different bacterial genera and even between different bacterial strains of the same genera. In Medium A, Ni uptake was observed to increase in the order *Methylobacterium* < *Paenibacillus* ≤ *Paraburkholderia* < *Pseudomonas*, with the average K_d_ values of 8.4676, 80, 81, and 676 L/kg DW in this medium, respectively. *Pseudomonas* had the highest average Ni uptake (average K_d_ 222 L/kg DW) also in Medium B. *Paraburkholderia* and *Paenibacillus* showed intermediate uptake of Ni(II) in Medium B, with *Paraburkholderia* showing generally higher Ni retention, with an average K_d_ of 64 L/kg DW, than *Paenibacillus* (average K_d_ 35 L/kg DW) in this growth medium. In Medium B, Ni removal by *Methylobacterium* was only marginal (average K_d_ 3.2 L/kg DW). The bacteria used in this study, had substantial variation in their substrate utilization patterns and metabolism, which presumably also affects their ability to remove Ni(II) under variable nutritional conditions. However, the ability of a strain to use a specific substrate, was not the most significant Ni(II) uptake controlling factor in the tested bacterial strains. *Pseudomonas* sp. PS-0-L and T5-6-I had very similar substrate utilization patterns, with only minor exceptions. *Pseudomonas* sp. V4-5-SB differed from these two strains in its ability to utilize different substrates, e.g., *Pseudomonas* sp. V4-5-SB had positive oxidase activity and was unable to utilize nitrate, while *Pseudomonas* sp. PS-0-L and T5-6-I were oxidase negative and capable of nitrate utilization. Nevertheless, the average Ni(II) uptake was at the same level for *Pseudomonas* sp. V4-5-SB and *Pseudomonas* sp. T5-6-I, while the average K_d_ values of *Pseudomonas* sp. PS-0-L differed substantially from the values of V4-5-SB and T5-6-I. *Pseudomonas* sp. PS-0-L had the highest Ni(II) uptake with an average K_d_ of 970 L/kg DW. In the other two *Pseudomonas* sp. strains a significantly lower average uptake, 240 L/kg DW, of Ni(II) was observed.

Because the highest Ni uptake was observed in *Pseudomonas* sp. V4-5-SB, PS-0-L and T5-6-I, these bacteria were selected for more detailed uptake studies. Solution pH has an important role in the metal uptake of bacteria, affecting both the metal speciation and solution chemistry, as well as surface chemistry of bacterial cell membranes and walls (Vijayaraghavan and Yun, 2008). As the pH increases, deprotonation of functional groups, such as carboxyl and phosphate groups, lead to the net negative charge of bacterial surfaces and additionally increase the electrochemical attraction and cation adsorption on bacterial cell membrane or cell wall (Jiang et al., 2004; Vijayaraghavan and Yun, 2008). In the present study, the effect of pH on Ni(II) removal by *Pseudomonas* sp. V4-5-SB, PS-0-L, and T5-6-I was examined at the pH range of 1.2–11.7. This pH range includes the natural pH range of most boreal soils and other environmental habitats (pH 6–9). On the other hand, at pH over 9, precipitation of metal species takes place, removing free metal cations from the solution and from the reach of the bacterial cells (Nieminen et al., 2007). In our research, the increase in the solution pH increased Ni(II) uptake by *Pseudomonas* sp. V4-5-SB, PS-0-L and T5-6-I, and the highest Ni uptake was observed in solution pH ∼6–9. As energy-independent interaction mechanisms, such as direct biosorption on cell wall functional groups, would be affected by pH, the observed increase in Ni(II) uptake with the increasing pH may indicate cationic Ni(II) biosorption on to the bacterial cell wall. Previously, the isoelectric points (IEP) of several Gram positive and Gram negative bacteria have been determined and the reported IEPs have varied from 1.75 to 4.15 (Harden and Harris, 1952). For *Pseudomonas cyanogenes* (strain 795), *Ps. convexa*, *Ps. aeruginosa* and *Ps. pyocyaneus*, IEP values between 2.2 and 3.3 have been reported (Winslow and Upton, 1926; Harden and Harris, 1952; Yee and Fein, 2001). These values are within the observed increase in the Ni uptake at pH range 2–4 in the current work, which indicates negative surface charge of *Pseudomonas* sp. V4-5-SB, PS-0-L, and T5-6-I at the pH above 4 and the cationic Ni(II) biosorption on to the bacterial cell wall. However, the observed Ni(II) uptake of *Pseudomonas* sp. V4-5-SB and PS-0-L were lower in 0.1 mol/L NaCl at pH ∼7 (K_d_ 502 and 807 L/kg DW) compared to the results in Medium A at + 20°C at pH 7 after 7 days incubation (K_d_ 640and 1890 L/kg DW). This may indicate, e.g., intra-cellular bioaccumulation of Ni or secretion of Ni-complexing metabolites by *Pseudomonas* sp. V4-5-SB and PS-0-L in addition to Ni(II) biosorption under these conditions.

The Ni(II) cell wall biosorption was further examined using adsorption isotherms. Adsorption isotherms are commonly used to describe the relation between the equilibrium concentration of an adsorbate [Ni(II)] and an adsorbent (bacterial cell) (Ho et al., 2002; Sparks, 2003). The use of isotherms requires their proper understanding and critical interpretation. Recently, linear regression analysis has generally been applied to determine the most suitable adsorption model because it analyzes the adsorption system and verifies the theoretical assumptions of isotherm model (Ayawei et al., 2017). However, the use of empirical models, e.g., Langmuir and Freundlich models, is limited and the previous studies cannot be directly applied to the systems obtained under conditions different or more complex than those studied. Generally, the shape of isotherms has been used to evaluate the capacity and affinity of sorption surface (Sparks, 2003). Isotherm shapes have also been used to estimate the number of different sorption sites of a particular surface taking part in the sorption process. We used both the Langmuir and Freundlich model equations to describe the absorption system in the two *Pseudomonas* sp. strains V4-5-SB and PS-0-L. Typically, the Langmuir model can be applied for sorption on monolayer surfaces (Wang and Chen, 2009). In this model, it is assumed that the metal ions are chemically adsorbed at a fixed number of well-defined sites; there is no interaction between the ions; all adsorption sites are energetically equivalent (homogeneous surface) and each site can accommodate one ion. However, the Langmuir model does not take into account that the surface is rarely homogeneous; the adsorbed ions are not necessarily inert, and the adsorption mechanism is clearly not the same for the first ions to adsorb to a surface as for the last one (Vijayaraghavan et al., 2006; Ayawei et al., 2017). Langmuir constants, q_m_ and b, represent sorption capacity and bonding energy of absorption (affinity), respectively (Wang and Chen, 2009; Oves et al., 2013). In general, low *b* values are reflected in the steep slope of a sorption isotherm, indicating a high affinity. Thus, for a good sorbent typically a high q_m_ value and a steep isotherm slope (i.e., low b) is observed (Wang and Chen, 2009). The experimental data of *Pseudomonas* sp. V4-5-SB and PS-0-L followed the Langmuir adsorption isotherm. The *b* value for *Pseudomonas* sp. V4-5-SB and PS-0-L at pH 5.7 were 468 and 46.3 L/g DW and the q_m_ values were 0.00494 and 0.0265 mg/g DW, respectively. At higher pH (8.7), the corresponding *b* values were 242 and 73.6 L/g DW and the q_m_ values were 0.00496 and 0.00756 mg/g DW. The Freundlich model can be applied for sorption on heterogeneous surfaces or surfaces supporting sites of varied affinities (multilayer sorption) (Volesky, 1990; Ho et al., 2002; Wang and Chen, 2009). This isotherm gives an expression which defines the surface heterogeneity and the exponential distribution of active sites (Ayawei et al., 2017). It is assumed that the stronger binding sites are occupied first and that the binding strength decreases with the increasing degree of binding site occupation (Vijayaraghavan et al., 2006). Biosorption equilibrium constant, K_f_, from the Freundlich sorption model represents sorption ability and n indicates biosorption intensity (Wang and Chen, 2009; Oves et al., 2013). However, K_f_ parameters cannot be compared among samples whenever the 1/n parameters differ (Chen et al., 1999). It is also not possible to compare K_f_ values from literature if they were derived from different units of q and C_e_ (Chen et al., 1999). Feundlich isotherm is not sufficient at very high concentrations of adsorbate because in reality the mass-ratio of adsorbate/adsorbent has an asymptotic maximum as concentration increases without limitations (Ayawei et al., 2017). Experimental sorption data of *Pseudomonas* sp. V4-5-SB and PS-0-L also followed, in addition to the Langmuir model, the Freundlich adsorption isotherm. The K_f_ value for *Pseudomonas* sp. V4-5-SB and PS-0-L at pH 5.7 were 0.221 and 0.403 mg L^1/n^ g^–1^ and the 1/n values were 0.763 and 0.876, respectively. At higher pH (8.7), the corresponding K_f_ values were 0.764 and 0.769 mg L^1/n^ g^–1^ and the 1/n values were 0.941 and 1.03. Both models, Freundlich and Langmuir, confirmed the higher sorption capacity and more intensive Ni(II) biosorption for *Pseudomonas* sp. PS-0-L compared to *Pseudomonas* sp. V4-5-SB.

Furthermore, the Freundlich adsorption isotherms of *Pseudomonas* sp. V4-5-SB and PS-0-L showed a slight deflection at both pH 5.7 and 8.7. At low initial Ni concentrations, isotherms were observed to behave ideally. Such behavior can be explained by the high affinity of the adsorbent for the adsorptive at lower Ni(II) concentrations and with decreasing affinity as the concentration increases. Additionally, the deflection of isotherms may result from the saturation of the exchange sites or from the retention of Ni(II) on multiple sorption sites. Generally, Freundlich sorption isotherms tend to fit experimental data better at low concentrations, while Langmuir isotherms fit the data better at higher concentrations (Richter et al., 1989). This was also observed in our experimental data. However, adsorption isotherms are descriptions of experimental data and more specific conclusions require support of molecular investigations, e.g., the use of spectroscopic techniques.

Ni biosorption over a range of metal ion concentrations with *Pseudomonas* sp. V4-5-SB and PS-0-L at pH 5.7 and 8.7 showed that the adsorption of Ni(II) was dependent on the initial Ni(II) concentration. The values of equilibrium uptake capacities (q) by *Pseudomonas* sp. V4-5-SB and PS-0-L increased as the pH of the solution and the amount of Ni(II) increased. The highest *q* values were achieved with the initial Ni(II) concentration of 10^–3^ mol/L. The *q* values by *Pseudomonas* sp. V4-5-SB and PS-0-L were 2.4 and 5.2 mg/g DW at pH 5.7, and 6.6 and 16.8 mg/g DW at pH 8.7, respectively. Previously, Ni(II) uptake has been studied with several bacteria, such as *E. coli*, *Pseudomonas* sp., *Bacillus* sp., *Arthrobacteria* sp., *Sterptomyces* sp., *Alcaligenes eutrophus*, under varying experimental conditions. The Ni biosorption capacities were reported to be between 0.8 and 55 mg/g DW at initial Ni concentrations of 10^–3^–10^–2^ mol/L (Mattuschka and Straube, 1993; Tsezos et al., 1995; Veglió et al., 1997; López et al., 2000; Öztürk et al., 2004; Selatnia et al., 2004; Ansari and Malik, 2007; Öztürk, 2007). In addition, a very high value of 100 mg/g DW by *Ps. fluorescens* 4F39 was reported, with initial Ni concentration of 10^–3^ mol/L at pH 9 (López et al., 2000). In our study, the Ni(II) uptake capacities fell in the middle of the typical uptake capacity range, being fivefold lower than the maximum capacity reported by López et al. (2000) for *Ps. fluorescens* 4F39. In our research, the highest Ni(II) uptake was shown by *Pseudomonas* sp. PS-0-L with the equilibrium Ni(II) biosorption capacity of approximately 17 mg/g DW at pH 8.7 with the initial Ni(II) concentration of 10^–3^ mol/L. However, all strains removed Ni(II) to a certain extent, implying that the capacity for Ni(II) uptake may be wide spread in soil microbial communities.

The role of cellular functional groups in the Ni(II) binding by *Pseudomonas* sp. V4-5-SB, PS-0-L, and T5-6-I was studied using FTIR spectroscopy. The functional groups found on *Pseudomonas* sp. cells were similar regardless of strain and all strains had alcoholic hydroxyl, amine, carboxylic carbonyl and hydroxyl, as well as phosphate and alkane groups. Of these groups, alcoholic hydroxyl, carboxylic hydroxyl and amine groups may participate Ni(II)binding, but as amine groups typically are protonated under physiological pH, the alcoholic and carboxylic hydroxyl groups most probably would act as the main Ni(II) binding groups in these bacterial cells, with lower sorption capacity found for the amino groups. This assumption is supported by the observations obtained using sorption isotherm approach, in which Ni biosorption and retention on multiple sorption sites was indicated. Previously, Choudhary and Sar (2009) have reported carboxyl, amide, and phosphate groups of *Pseudomonas* sp. J007 for being the dominant functional groups involved in bacteria-metal (Ni, Co, Cu, Cd) interactions. In addition, the presence of carboxyl and amine groups on the surfaces of *Ps. aeruginosa* (Kang et al., 2007) and carboxyl and phosphate groups on *Pseudomonas* sp. MTCC 3087 (Kazy et al., 2006) have been reported. Further, Yu et al. (2014) have suggested that sulfhydryl groups (-SH) may also control the binding of metals onto bacterial walls under low metal-loading conditions.

We studied the morphology of *Pseudomonas* sp. V4-5-SB and PS-0-L in Ni(II) treated cells using TEM. As stated above, López et al. (2000) have reported accumulation of dense Ni structures on cell walls of *Ps. fluorescens* 4F39 at pH 9. Additionally, *Ps. aeruginosa* has been shown to bind Ni onto the cell envelope region (Sar et al., 2001). On the basis of these previous observation on *Ps. fluorescens* and *Ps. aeruginosa* and on our results using FTIR, we assumed that Ni(II) attached to the bacterial cell membrane or cell wall. However, TEM images showed dense intra-cellular Ni accumulations in the cells of *Pseudomonas* sp. V4-5-SB and PS-0-L treated with Ni(II), but no significant Ni deposits were observed on or in the close vicinity of the cell membrane/cell wall of these bacteria. EDS analysis confirmed the presence of the Ni in these bacteria although at low concentrations. To our knowledge, clear Ni structures/crystals inside the bacterial cells or in cytoplasm have not been reported previously. However, copper (Cu^2+^) and cadmium (Cd^2+^) uptake have been detected in and around the cell periphery of *Pseudomonas* sp. J007, with a small fraction of metal in the cytoplasm (initial metal conc. 5 × 10^–4^ mol/L) (Choudhary and Sar, 2009). In addition, fractionation experiments showed low nickel (Ni^2+^) distribution in the cytoplasm (Choudhary and Sar, 2009). Divalent heavy metal cations are structurally very similar (radius, charge) and are expected to behave in a similar way, supporting our finding of Ni(II) accumulations inside bacterial cells. It is also known that several bacteria have uptake systems for Ni and other heavy metals (Nies, 1999; Eitinger and Mandrand-Berthelot, 2000). For example, Smith and Maguire (1995) identified CorA Mg^2+^ transporter system (metal inorganic transport) in several Gram negative bacteria. In addition to magnesium, it has been suggested that CorA also mediates uptake of Ni^2+^ and Co^2+^ (Snavely et al., 1989, 1991). Certain ABC-type transporters and transporters of the HoxN family have been identified to possibly convey different divalent cations (Nies, 1999). The Ni crystals observed in the TEM images of our study, may indicate active uptake of Ni(II). This was also supported by the several changes observed in the protein expression profiles of these bacteria. However, the relatively high Ni concentrations used in our study, may have activated the detoxification mechanism in the bacterial cells. This mechanism could be further utilized for bio-purification applications in contaminated sites.

We used the incubation temperatures of +4 and +20°C in our experiments and + 20°C was chosen to represent the average daytime surface temperatures found in southwestern Finland in June (Finnish Meteorological Institute [FMI], 2015). The lower temperature was chosen to represent the temperature of the bottom layers of the bog. All bacterial strains used in this study removed Ni(II) from the solution more efficiently at + 20°C. The average uptake at this temperature was 89% higher than the average uptake observed at + 4°C. Similarly, at this temperature, the bacterial growth rate was higher compared to that at the lower temperature of + 4°C, which is likely to affect the efficiency of Ni(II) removal by biosorption and/or bioaccumulation as the cell densities at the time = 0 were taken into account in the calculations. Moreover, the presence of Ni(II) (10^–6^–10^–3^ mol/L) was not found to affect bacterial growth, indicating that the possible toxic effect of Ni(II) is either surpassed by the bacterial metabolism (e.g., precipitation of Ni inside the cells through detoxification reactions) or the used Ni(II) concentrations were below the toxic levels. Growing bacteria may accumulate Ni inside the cell as part of their metabolism or release metabolites into the solution that may affect Ni solubility. In addition, the increasing amount of biomass provides more surface area and functional groups for Ni(II) biosorption to potentially occur on cell surfaces.

In addition to the effect of temperature on cell growth rate, incubation time affects the growth phase of bacteria. After the exponential growth phase, the biomass typically reaches a plateau phase in which growth rate and death rate are equal. After a certain period of time, the amount of bacterial mass begins to decrease and cell death prevails (death phase). In the current research, Ni(II) uptake varied with different bacterial strains depending on the incubation time. As we increased the incubation time from 7 to 14 days, we observed 43% decrease in Ni(II) uptake by the studied bacteria, which may result from increased cell death and cell lysis in the cultures. This supports the active Ni(II) uptake hypothesis for the bacteria used in the present study, as Ni is potentially released from the cells after cell lysis, resulting in decreased overall uptake. If direct biosorption on cell walls would occur, decrease after cell lysis would not be expected as biosorption occurs between the absorbent and cell wall functional groups regardless of whether the cells are alive or dead. Previously, we studied interaction of Ni(II) with other bacterial isolates (*Pseudomonas, Paraburkholderia, Rhodococcus, Paenibacillus*) from same Lastensuo bog (Lusa et al., 2016a) and observed that Ni(II) uptake to increase as the incubation time was increased from 1–3 to 7–14 days, which suggested that the 7-day incubation time is optimal, especially in the case of *Pseudomonas* sp. PS-0-L and T5-6-I, which is in line with the results obtained also in the current study.

Our results obtained from the batch uptake studies under different nutrient conditions, temperature and pH as well as the spectroscopic data using EDS and finally the data obtained using electron microscopy (TEM) all supported Ni accumulation inside the bacterial cells used in this study. In addition, several changes in the protein profiles in the bacteria treated with Ni(II) were observed in the MALDI-TOFF spectra, which can indicate active accumulation of Ni in these bacteria. However, at the same time the uptake of Ni(II) was observed to follow the Langmuir and Freundlich isotherm models, which are typically used to describe the direct sorption (or in our case biosorption). The sorption isotherms indicated Ni(II) retention on multiple sorption sites. In addition, FTIR analysis showed the presence of the alcoholic hydroxyl, carboxylic carbonyl and hydroxyl, amine, alkane, and phosphate groups on bacterial surfaces. Based on these results we propose a distinct uptake mechanisms of Ni(II) in the *Pseudomonas* sp. strains used in this work; (i) intra-cellular accumulation (e.g., through detoxification mechanism) and (ii) direct biosorption on cell membrane or wall functional groups. Even though the proportions of Ni(II) removed from the solution by bioaccumulation and biosorption could not be fully concluded in our study, the fact that Ni was detected inside the bacterial cells, and that the sorption isotherm model could (at least partly) describe the uptake process implies in two different uptake mechanisms present in these bacteria.

## Conclusion

All studied bacteria were able to remove Ni(II) from the solution and the uptake was affected by incubation time, temperature, pH as well as nutrient solution used. Temperature, incubation time, and nutrient source also affected the overall bacterial growth rate, affecting presumably also Ni(II) removal. Even though the isolated bacteria belonged to the minority of the whole bacterial population of the bog, the fact that they all were able to remove Ni(II) from solution indicates that this is a common feature for bacteria found in this environment and that it is most likely that soil bacteria are capable to influence the biogeochemical behavior of Ni(II) in the northern, boreal environment also under *in situ* conditions. Based on our ^63^Ni batch uptake experiments as well as the results obtained from EDS, TEM, FTIR, and MALDI-TOFF studies, we suggest that in the studied bacterial strains (especially the *Pseudomonas* sp. strains), Ni(II) is taken up inside the cells using an active mechanisms and in addition, part of the Ni(II) is retained on the cell membrane/cell wall functional groups through biosorption.

## Data Availability Statement

The datasets generated for this study can be found in NCBI GenBank, accession numbers MK290398–MK290403.

## Author Contributions

JK and ML participated in the design of the study. JK isolated new bacterial strains, characterized them and extracted DNA, performed all biosorption experiments, transmission electron microscopy (TEM), and Fourier transform infrared (FTIR) analyses, carried out the statistical analyses, and prepared the morphological samples. ML and MB performed a phylogenetic analysis and wrote this part of the manuscript and supervised the project. MK characterized the morphological samples with Energy dispersive X-ray spectroscopy (EDS). JK wrote the manuscript with support from ML and MB. All authors contributed to the interpretation of the results, provided critical feedback and contributed to the final manuscript.

## Conflict of Interest

MB is employed by the company VTT Technical Research Centre of Finland, Ltd.

The remaining authors declare that the research was conducted in the absence of any commercial or financial relationships that could be construed as a potential conflict of interest.
