# Supplementary material for: Ni(II) Interactions in Boreal Paenibacillus sp., Methylobacterium sp., Paraburkholderia sp., and Pseudomonas sp. Strains Isolated From an Acidic, Ombrotrophic Bog
Source: Front Microbiol. 2019 Nov 26;10:2677. doi: 10.3389/fmicb.2019.02677 (PMC6901981; doi:10.3389/fmicb.2019.02677)
Supplement: Supplementary file 1 [file Image_1.pdf]

Supplementary Figure:

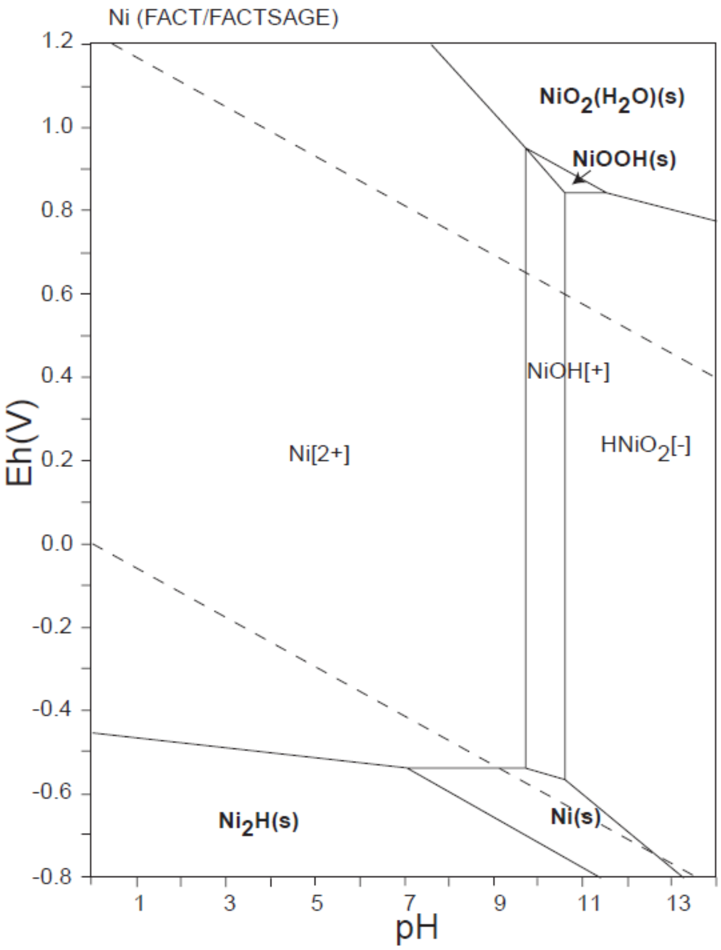

Supplementary Figure 1. Eh-pH diagram of nickel ($\Sigma Ni = 10^{-10}$, 298.15 K, 10^5 Pa) (Atlas of Eh-pH diagrams, Geological Survey of Japan Open File Report No. 419, 2005).
